# Accessing and re-accessing mental health walk-in clinics for children and families

**DOI:** 10.1177/27550834231200617

**Published:** 2023-09-29

**Authors:** Catalina Sarmiento, Graham J. Reid

**Affiliations:** 1 Department of Psychology, The University of Western Ontario, London, ON, Canada; 2 Departments of Psychology, Family Medicine, and Paediatrics, The University of Western Ontario, London, ON, Canada; 3 Children’s Health Research Institute, London, ON, Canada

**Keywords:** Mental health walk-in clinics, children, youth, Ontario, Canada

## Abstract

**Background:**

Many child and youth mental health (CYMH) agencies across Canada and in Ontario are using mental health walk-in clinics (MHWCs).

**Objectives:**

(1) Explore how MHWCs are used by families (e.g. mean, mode, and median number of visits), and (2) document how often and how soon families returned for a second MHWC visit and identify correlates of time to a second MHWC visit.

**Design:**

Administrative data from two CYMH agencies in Ontario were extracted, including demographics, visit data, and presenting concerns.

**Methods:**

In this exploratory, descriptive study, analyses of administrative data were conducted to identify patterns and correlates of MHWC use before other agency services, compared to MHWC use exclusively.

**Results:**

About a third of children and families using MHWCs had two or more visits. Child age, guardianship, and disposition at discharge emerged as correlates of time to a second MHWC visit.

**Conclusion:**

MHWCs can save families’ time, and both agencies’ time and money by eliminating the need to complete a detailed assessment prior to treatment for cases that would go on to have a single visit within this service.

## Introduction

Despite the high and growing need for help, only 26%–34% of children with mental health problems receive specialized mental health services.^1,2^ One of the main barriers to accessing mental health services are long wait lists. Children’s Mental Health Ontario,^3^ for example, reports that waitlists have continued to grow, and 28,000 children and youth wait as long as 2.5 years for services. These concerning statistics have led researchers and policy-makers to call for changes to service delivery.^1,4^ Scheduled single session therapy (SST) and unscheduled SST (i.e. mental health walk-in clinics [MHWCs]) are two forms of brief therapy that have gained attention and traction. These service delivery models treat each session as if it will be the only session with a client.^5^ Each session has the three main components of a complete therapy: (a) getting to know the client; (b) exploring the issue, strengths, and resources; and (c) developing a plan (e.g. strategies to implement).^6^

Satisfaction with scheduled and unscheduled SST services has been high.^6–10^ Generally, scheduled SST has been studied more extensively than MHWCs, which has allowed for literature reviews and meta-analyses. These have supported the use of scheduled SST for a number of presenting concerns, including anxiety, conduct problems, self-esteem, substance use, and general distress.^5,11^ Research on MHWCs is much more limited. The following sections briefly review this literature, followed by the theoretical framework used in this study.

### MHWC service use

MHWCs differ from scheduled SST in that they remove administrative hassles for families, including making phone calls, scheduling appointments, attending intakes, and waiting days to weeks for the service.^6,12^ A recent provincial survey of child and youth mental health (CYMH) agencies in Ontario examined the implementation of MHWCs in the province.^13^ The modalities (e.g. consulting break, outsider witness) and approaches (e.g. narrative therapy, solution-focused, therapy, and cognitive behavioral therapy) used were found to be flexible and varied. Social workers and registered psychotherapists were the most common providers of this service. MHWCs were provided from more than one location (e.g. agency, school community center, doctor’s office), likely making it easier for families to access.

The limited research on MHWCs suggests perceived improvement in level of distress, confidence to address the problem, and hopefulness immediately after the session;^8,14^ and improvement at follow-up on psychopathology, impairment, and distress.^8,15,16^ How families use MHWCs is also important to understand. When MHWCs are available, how often are they being used? If families do use an MHWC, does one session appear to have met their needs, or do they return shortly thereafter for another visit? Past research on MHWCs has found that 44%–50% of families reported that a single session addressed their concerns.^4,10^ This is similar to scheduled SSTs, where 50% of clients decide that a single session is adequate and do not schedule/attend further sessions.^17^ These data are from post-session surveys, and we do not know how often families are using this service. To address this gap in the literature, we used administrative data which does not rely on client reports. The treatment approach used in MHWCs assumes that a single session will be sufficient to meet families’ needs. However, if a high percentage of families return for a second MHWC visit within a short period of time, this could suggest that a change in the conceptualization of this service is needed.

If families do return for a second MHWC visit, it would be important to know how soon they return and who is returning for services. First, repeated MHWC visits close in time might indicate unmet needs. On the contrary, re-accessing an MHWC months later might be better viewed as seeking service when needed. Second, knowing the characteristics of who does versus does not return (suggesting their needs were met) might help in directing the families most likely to benefit to an MHWC. However, correlates of having a second MHWC visit are unknown. As such, correlates of accessing and re-accessing outpatient mental health services are reviewed. We focused on social content correlates, as these are typically the only variables consistently available in administrative data sets.

### Correlates of accessing and re-accessing services

Studies examining age, sex, and socio-economic status as correlates have yielded contradictory results.^18–28^ Specifically, the significance and direction of these relationships differs across studies. Single-parent households^18,21,25,26^ as well as problem severity and persistence^18,29^ appear to predict accessing services. Associations with externalizing and internalizing problems and impairments are mixed.^18,19,22,29^

Only one previous study has examined re-accessing outpatient services. Conducted in Ontario, it is found that younger children and families with unknown parental marital status had an increased risk while single-parent households had a decreased risk of re-accessing services after an episode of care.^30^

### Theoretical framework

The Revised Network-Episode Model^31^ provides a descriptive outline of four broad factors (and 76 nested variables), and the relationship between these factors that are thought to have direct and interactive effects on service use. Applying this model to MHWCs suggests that social content factors, such as characteristics of the child, parent, and family (e.g. child age, child gender, presenting concern, parental marital status) and illness career factors, such as key exits (e.g. termination of care, referral) may impact service use. As such, these variables were examined in this study.

### Current study

This study had two objectives: (1) explore how families use MHWCs (e.g. mean, mode, median number of visits), and (2) document how often and how soon families returned for a second MHWC visit and identify correlates of time to a second MHWC visit. Given the mixed findings in the service use literature and limited research on MHWCs, social content (e.g. child age, child gender, guardianship, neighborhood poverty, presenting concerns) and illness career (e.g. disposition at discharge) variables were explored, but specific hypotheses for these correlates were not made.

## Methods

The study was conducted in Ontario, Canada. As this is the first study to examine how MHWCs are used by families using administrative data from CYMH agencies, an exploratory, descriptive methodology was used.^32,33^ In the absence of similar studies in the literature, it was not possible to conduct a-priori power/sample size calculations.

### Inclusion and exclusion criteria for agencies

The mental health service network for children and families in Ontario is complex. This is because children and families can receive services from different sectors, including health (e.g. CYMH agencies, family health teams, hospitals, pediatricians, and psychiatrists), education, child welfare, juvenile justice, and private (e.g. psychologists, social workers) sectors. The pathway to access these services varies (e.g. self-referral for CYMH agencies; family physician referral for pediatricians and psychiatrists) as does the funding/cost (e.g. publicly funded CYMH agencies; fee-for-service psychologists and social workers).^34^ It is also important to note that there are significant differences in the service mandates even within the same sector and type of mental health provider. For example, CYMH agencies in the health sector can serve the entire community (e.g. population living within a delimited geographic area) or a subset of the community (e.g. subset of a population, like First Nations, living within a given geographic area), and children of all ages (i.e. birth to 18 years) or a subset of children (e.g. birth to 12 years). An agency can provide services for most mental health concerns (e.g. anxiety, depression, attention, hyperactivity, and non-compliance) or only for specific concerns (e.g. addictions only). Services may also be offered in a variety of different formats/settings (e.g. phone *and* face-to-face; outpatient *and* residential) or only in one format/setting (e.g. phone only; residential care only).

This study sought to identify CYMH agencies: serving all children (i.e. birth to 18 years) in the entire community (i.e. population living within a given geographic area), providing services for most mental health concerns (e.g. anxiety, depression, attention, hyperactivity, and non-compliance), delivering services in a range of formats and settings (e.g. phone *and* face-to-face; outpatient *and* residential), and located in a census division with a small urban center. This size for urban centers^35^ was selected for two main reasons. First, the population is large enough to yield sufficient data. Second, previous work has demonstrated that large urban areas in Ontario, metropolitan Toronto/Greater Toronto Area, in particular, have more CYMH agencies in a given area and higher prevalence of children and youth using services.^36^ As such, families in these areas may receive services from multiple agencies, which would be difficult to account for. This would bias the results as some families could experience the event of interest (i.e. a second MHWC visit), but they would be censored.

The inclusion criteria for agencies were as follows: (a) serve children birth to 18 years, (b) no fees for mental health services, (c) providing face-to-face services (pre-COVID-19 pandemic), (d) providing MHWCs before 2020 (i.e. pre-COVID-19 pandemic), and (e) located in a census division with a small urban center (population between 50,000 and 200,000).

The exclusion criteria for agencies were as follows: (a) primarily focus on specific disorders (e.g. addictions, developmental disorders, disabilities, bereavement, palliative care, health, criminality/justice system), (b) provides only informal supports (e.g. peer support), (c) provides only non-mental health services (e.g. employment, housing), (d) does not provide outpatient services (i.e. only residential or day treatment), and (e) serving only a specific subset of the community (e.g. LGBTQ, First Nations/indigenous).

#### Agency recruitment

CYMH agencies in Ontario that completed a provincial survey^13^ examining the implementation of MHWCs across the province were considered for this study. A total of 11 agencies met the eligibility criteria and were invited to participate via email. All agencies responded to this invitation, and a videoconference was scheduled with those interested in the study. Seven agencies declined to participate; the most common reason for declining was limited capacity (e.g. time and resources). Four agencies agreed to participate. Two of the agencies had data that could not be used. One implemented the MHWCs in 2019 and had a small sample of MHWC clients. The other had separate electronic administrative records for MHWC clients and other agency clients; these records, unfortunately, could not be reliably linked. The remaining two agencies had data that could be used for the study.

Electronic administrative data were extracted and de-identified by EMHware personnel. EMHware is a company that produces and manages electronic record systems for many CYMH agencies in Ontario. Agencies varied in when they started using EMHware and whether and how previous data were migrated. Because of this, the window of data availability varied across agencies. In addition, COVID-19 pandemic resulted in a lockdown announcement in Ontario on 17 March 2020, leading to a halt to all in-person services. As a result, only data prior to 17 March 2020 were analyzed.

#### Participating agencies

Agency 1 is located in southwestern Ontario. They implemented their MHWCs in 2013, and electronic data were available since 2008. The MHWCs in Agency 1 are offered from different locations (e.g. agency, community center, physician office) by child and youth workers, social workers, and registered psychotherapists. Different approaches are used, including narrative therapy, solution-focused therapy, supportive therapy, and other (e.g. emotion focused therapy, dialectical behavior therapy skills, motivational interviewing). In this agency, MHWCs were either unscheduled (estimated 85%–90%) or scheduled (estimated 10%–15%) single session appointments (before COVID-19; personal communication, Agency 1, 2021) and served as a point of intake for agency services (i.e. gathering information, such as presenting problem, to decide what services are offered). Of note, families can complete this intake in other ways (e.g. phone calls). The study window for Agency 1 was 6 years and 9 months from June 2013 to March 2020.

Agency 2 is located in southeastern Ontario. They implemented MHWCs in 2006, and electronic data were available since 2016. The MHWCs in Agency 2 are offered at their agency by social workers and registered psychotherapists. Different approaches are used, including narrative therapy, solution-focused therapy, and choice and partnership approach. In this agency, MHWCs were either unscheduled (estimated 85%–90%) or scheduled (estimated 10%–15%) single session appointments (before COVID-19) and did not serve as a point of intake for agency services (personal communication, Agency 2, 2021). The study window was 3 years and 8 months from June 2016 to March 2020.

#### Inclusion and exclusion criteria for agency clients

The inclusion criteria for children and families were as follows: (a) had an MHWC visit; (b) children under the age of 16 at the start of the study window, ensuring that they were able to access agency services; and (c) children under the age of 16 at their first MHWC visit, allowing families the opportunity to re-access MHWCs even if they first accessed the service in the second half of the study window.

**Figure 1.**
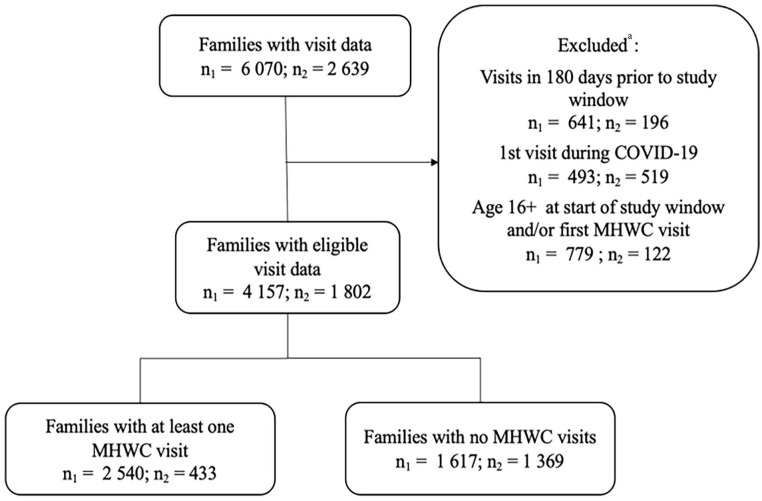
Flow chart showing children/family selection for Agency 1 (*n*_1_) and Agency 2 (*n*_2_). ^a^Criteria applied sequentially in the order shown. MHWC: mental health walk-in clinic.

The exclusion criteria for children and families were as follows: (a) children who had visits in the 180 days prior to the study window; thus, all cases included would be starting a new episode of care^37^; (b) cases with phone contact only (i.e. no face-to-face or videoconference contacts); and (c) cases where a parent of a child above 12 years old accessed services *without* their child present. This is because children above 12 years old must consent to have a file opened for them, otherwise no identifiable information is recorded in the database. Figure 1 presents the sample selection.

### Electronic administrative data

Each contact that agency personnel had with the child/family (or a school or other professional for the purposes of delivering and coordinating services) is recorded in the electronic records. This information is entered by agency staff (e.g. clinicians, intake workers) shortly after each contact. The information entered for the first MHWC visit was utilized as correlates. Only non-identifying information were abstracted from the electronic administrative database. Variables abstracted and analyzed are presented below.

#### Outcomes

We computed the percentage of families having at least one MHWC visit using data from all eligible cases (See Figure 1). Among families with at least one MHWC visit, we computed descriptives for the number of visits and time to a second visit (i.e. re-accessing). The outcome, time to re-access MHWC services, was operationally defined as time in days between the first MHWC visit and the second MHWC visit.

#### Child age

The age (years) at the time of the first MHWC visit was calculated for each child. It was re-coded as a categorical correlate with two categories: <12 years old, and 12+ years old.

#### Child gender

The agencies entered child gender using three categories: female, male, and other. The “other” gender category had small cell sizes. As such, it was included in descriptive statistics, but these cases were dropped (*n*_Agency 1_ = 19; *n*_Agency 2_ = 2) in the Cox regressions.

#### Neighborhood poverty

As family income was not available, neighborhood data derived from matching the family’s postal code to census data were used. The following steps were taken: (1) the prevalence of low-income households (using the Low-Income Measure) was obtained for each forward sortation area (FSA; i.e., first three digits of the postal code) from the 2016 Census^38^; (2) each FSA was categorized into two quantiles: high poverty (i.e. LIM 12.7 to 47.7; mean = 19.59; SD = 6.11) or low poverty (i.e. LIM 3.1 to 12.6; mean = 8.66; SD = 2.52); and (3) the neighborhood poverty two-quantile was assigned to each case based on the family’s postal code.

#### Guardianship

The agencies entered guardianship using seven categories: birth/adoptive father, birth/adoptive mother, birth/adoptive parents, shared custody, grandparents, Children’s Aid Society (i.e. child welfare), and other. Note that shared custody could range from equal custody between parents to predominately one parent. Parents’ relationship status was not available; thus, guardianship with birth/adoptive father or birth/adoptive mother, may or may not have reflected a single-parent family. Due to small cell sizes, it was re-coded into four categories: birth/adoptive parents, shared custody, birth/adoptive father or mother, and other.

#### Presenting concern

The agencies entered presenting concerns from a list of more than 50 options with only minor differences between agencies; coding of multiple concerns was possible. Presenting concern for the first MHWC visit was re-coded into four categories: (1) externalizing concerns, (2) internalizing concerns, (3) parenting and family concerns, and (4) other concerns (e.g. school problems, sleep difficulties). As multiple problems were often coded, four variables were computed and coded as: not a concern or was a concern.

In addition, the total number of presenting concern categories (out of 4) was computed for each visit and used as a proxy for complexity and comorbidity. Due to small cell sizes, it was re-coded as a categorical correlate with three categories: (1) presenting concern category, (2) presenting concern categories, and (3+) presenting concern categories.

#### Disposition at discharge

The agencies entered disposition at discharge after the first MHWC visit using six categories. It was re-coded as a categorical correlate with two broad categories: (1) no referral within the agency: no referral was made to services within the agency *or* referral was made to services outside of the agency (rare; 0.04% of sample); and (2) referral within the agency: anticipate seeing the family again at the agency (see Supplemental Table S1 for more information). Of note, this variable could only be explored with Agency 1.

### Data analyses

Analyses were conducted in SPSS (Version 27) for Windows. All analyses were conducted separately for each agency, given differences in how MHWCs were used, when they were implemented, and number of years of data provided.

Continuous survival analyses, Cox regressions, were used to identify factors that influence time to a second MHWC visit. Unadjusted and adjusted hazards ratios (HRs) were calculated to determine the effect that each correlate had on the outcome independently and adjusting for other variables. HRs can be interpreted as the change in risk of a second MHWC visit for every one-unit increase (e.g. 1 year increase in age) or compared to another category (e.g. males compared to females).

Correlates were entered in blocks based on the Revised Network-Episode Model categories.^31^ The overall model was interpreted first, follow by the individual correlates. Cox regressions were used as it accounts for censoring (i.e. some participants do not experience the event of interest).^39^

#### Assumptions

The key assumptions for a Cox regression are proportional hazards and non-informative censoring.^39^ Proportional hazards specify that the HR for each correlate is constant over time. Time-dependent covariates (i.e. interaction of each correlate with time) were examined to evaluate the proportional hazard assumption.

Non-informative censoring stipulates that there should not be a correlation between time-to-event and time of censoring. In this study, this might have occurred if families sought other MHWC services (e.g. private provider). This is acknowledged as a potential source of bias in estimates.^39,40^

#### Missing data

Multiple imputation (40 imputations) was used to handle missing data for the correlates used in the Cox regressions. Multiple imputation was conducted separately for each agency. Overall, 69.0% and 68.0% of families in Agency 1 and Agency 2, respectively, had no missing data across correlates (see Supplemental Table S2 for more information).

##### Results

Table 1 presents the descriptive statistics for all families with an MHWC visit prior to multiple imputations. Between 36.8% and 39.0% of the sample was age 12 and above, and between 42.3% and 50.0% were females. There were statistically significant differences between the two agencies in all variables, except for child’s age.

**Table 1.** Descriptive statistics of child, family, and service use for all families with an MHWC visit.

	Agency 1 (*N* = 2540)	Agency 2 (*N* = 433)
	*n* (%)	*n* (%)
Child		
Child age		
<12 years old	1606 (63.2%)	264 (61.0%)
12+ years old	934 (36.8%)	169 (39.0%)
Child gender		
Female	1271 (50.0%)*	183 (42.3%)*
Male	1248 (49.1%)	223 (51.5%)
Other	19 (0.7%)	2 (0.5%)
Missing	2 (0.1%)*	25 (5.8%)*
Family		
Guardianship of child		
Birth/adoptive parents	812 (32.0%)	150 (36.7%)
Birth/adoptive mother	591 (23.3%)	113 (26.1%)
Birth/adoptive father	101 (4.0%)	20 (4.6%)
Shared custody	425 (16.7%)*	45 (10.4%)*
Grandparents	85 (3.3%)	20 (4.6%)
Children’s Aid Society	30 (1.2%)	6 (1.4%)
Other	25 (1.0%)	6 (1.4%)
Missing	471 (18.5%)	64 (14.8%)
Neighborhood poverty		
Low poverty	1789 (70.4%)*	259 (59.8%)*
High poverty	733 (28.9%)*	161 (37.2%)*
Missing	18 (0.7%)*	13 (3.0%)*
Service use		
Presenting concern^a^		
Externalizing	806 (31.7%)	171 (39.5%)
Internalizing	1292 (50.9%)*	236 (54.5%)*
Parenting and family	639 (25.2%)*	57 (13.2%)*
Other	500 (19.7%)*	236 (54.5%)*
Missing	717 (28.2%)*	51 (11.8%)*
Number of presenting concerns		
1	786 (30.9%)	161 (37.2%)
2	725 (28.5%)	136 (31.4%)
3	247 (9.7%)*	73 (16.9%)*
4	65 (2.6%)	12 (2.8%)
Missing	717 (28.2%)*	51 (11.8%)*
Disposition at discharge		
No referral within the agency	1517 (59.7%)	N/A
Referral within the agency	961 (37.8%)	N/A
Missing	62 (2.4%)	N/A

Descriptive statistics between the two agencies were compared using chi-square tests and *z* pairwise tests (if chi-square tests was significant to determine which proportion was different).

MHWC: mental health walk-in clinic.

a Clinicians could code multiple presenting concerns for a visit.

* *p* < .05

### Use of MHWCs

For Agency 1, 61.1% of families with eligible visit data had at least one MHWC visit. Of the families, 38% were referred within the agency for other services (See Supplemental Table S1). The number of MHWC visits a family had ranged from 1 to 10 with a mean of 1.50 (SD = 0.91), median of 1, and mode of 1; and 32.3% of these families had two or more visits. For Agency 2, 24.0% of families with eligible visit data had an MHWC visit. The number of MHWC visits a family had ranged from 1 to 8 with a mean of 1.70 (SD = 1.27), median of 1, and mode of 1; and 36.3%% of these families had two or more MHWC visits. The difference in the number of MHWC visits was statistically significant across the agencies (*U* = 579089, *p* < .05); however, for both agencies, the median and mode were the same (see Supplemental Table S3).

#### Having a second MHWC visit

For Agency 1, time to a second MHWC visit ranged from 1 to 2,198 days with a mean of 412.77 days (*SD* = 432.39) and median of 259 days. For Agency 2, time to a second MHWC visit ranged from 1 to 917 days with a mean of 111.72 days (*SD* = 188.86) and median of 21 days. The difference in mean time to a second MHWC visit was statistically different (*U* = 31078, *p* < .01) across the agencies. Supplemental Figure S1 shows the survival probability by time (days).

For Agency 1, the full Cox regression model predicting time to a second visit provided an adequate fit based on the omnibus test (*p* < .05). The social content block and illness career block each independently provided an adequate fit. In the multivariate model, children <12 years old had 18% higher risk of a second MHWC visit, compared to children 12+ years old; children in shared custody had 33% higher risk of a second MHWC visit and children under the guardianship of birth/adoptive mother or father had 31% higher risk of a second MHWC visit, compared to children under the guardianship of birth/adoptive parents; and children with a disposition at discharge coded as “no referral within the agency” had 50% higher risk of a second MHWC visit, compared to those with a disposition at discharge coded as “referral within the agency” (see Table 2).

**Table 2.** Unadjusted and adjusted hazards ratios for time to a second visit.

	Agency 1		Agency 2	
	Unadjusted	Adjusted	Unadjusted	Adjusted
	HR (95% CI)	HR (95% CI)	HR (95% CI)	HR (95% CI)
Social content				
Child age^a^				
<12 years old	1.18 (1.02–1.37)*	1.18 (1.01–1.37)*	1.20 (0.86–1.67)	1.21 (0.85–1.72)
Child gender^b^				
Male	1.04 (0.90–1.19)	1.01 (0.86–1.17)	0.79 (0.57–1.11)	0.75 (0.52–1.07)
Neighborhood poverty^c^				
High poverty	1.17 (1.01–1.36)*	1.13 (0.97–1.33)	0.84 (0.60–1.18)	0.81 (0.57–1.14)
Guardianship of child^d^				
Shared custody	1.32 (1.07–1.63)*	1.33 (1.04–1.69)*	1.20 (0.71–2.03)	1.23 (0.72–2.10)
Birth/adoptive mother or father	1.29 (1.07–1.55)**	1.31 (1.07–1.60)*	0.85 (0.58–1.25)	0.83 (0.55–1.24)
Other	1.22 (0.90–1.65)	1.23 (0.88–1.72)	1.01 (0.55–1.86)	1.02 (0.54–1.91)
Presenting concern				
Externalizing^e^	0.98 (0.82–1.18)	1.02 (0.75–1.37)	0.94 (0.67–1.32)	1.04 (0.52–2.08)
Internalizing^e^	0.99 (0.84–1.19)	1.09 (0.82–1.45)	0.99 (0.71–1.41)	1.05 (0.54–2.05)
Parenting and family^e^	1.01 (0.84–1.21)	0.95 (0.70–1.29)	0.96 (0.59–1.56)	1.04 (0.51–2.12)
Other^e^	0.97 (0.80–1.18)	0.97 (0.71–1.33)	1.03 (0.73–1.46)	1.02 (0.52–1.99)
Number of presenting concerns^f^				
1	1.04 (0.80–1.35)	1.10 (0.64–1.88)	1.13 (0.71–1.79)	1.22 (0.34–4.34)
2	1.04 (0.81–1.34)	1.05 (0.73–1.52)	1.28 (0.79–2.07)	1.39 (0.64–3.05)
Illness career				
Disposition at discharge^g^				
No referral within the agency	1.41 (1.20–1.65)**	1.50 (1.26–1.78)**	N/A	N/A

HR: hazards ratio; CI: confidence interval.

a Reference category is children aged 12+.

b Reference category is females.

c Reference category is low poverty.

d Reference category is birth/adoptive parents.

e Reference category is no presenting problem in that category.

f Reference category 3+ presenting concern categories.

g Reference category is referral within the agency.

* *p* < 0.05; **p < 0.01.

This model was re-run without disposition at discharge to be better able to compare the findings to Agency 2 (see Supplemental Table S4). The were no substantial differences in the results.

For Agency 2, the full Cox regression model predicting time to a second visit did not provide an adequate fit based on the omnibus test (*p* > .05). Of note, only the social content block could be tested due to data availability. No adjusted or unadjusted correlates were significant.

Descriptive statistics for the subsample used in the regressions following multiple imputation procedures are presented in the Supplemental Table S5.

### Supplementary analyses

Age, guardianship, and disposition at discharge were significant correlates of time to a second visit. It is possible that there are differences with respect to presenting concerns within these subgroups (e.g. younger children have more externalizing problems, whereas older children have more internalizing problems). As such, these differences were explored. Analyses were only conducted with data from Agency 1, as there were no significant correlates for Agency 2.

Children < 12 years old had more externalizing problems whereas children 12+ years old had significantly more internalizing problems. Children in shared custody had more parenting and family problems than children under the guardianship of birth/adoptive parents. Children under the guardianship of a birth/adoptive father or mother had less internalizing problems, and more parenting and family problems than children under the guardianship of birth/adoptive parents. Finally, children with a disposition at discharge coded as “referral within the agency” had more internalizing problems, whereas children with a disposition at discharge coded as “no referral within the agency” had more parenting and family problems (see Tables S6a, S6b, and S6c).

## Discussion

This study examined MHWC use in two CYMH agencies. The first objective was to explore how MHWCs are used by families. The percentage of all agency clients using the MHWCs was different across the agencies: 61.1% in Agency 1 versus 24.0% in Agency 2. This difference likely stemmed from the way in which the MHWCs were implemented. Specifically, Agency 1 uses it as a point of intake, while Agency 2 does not. As such, it is not surprising that 38% of the MHWC clients at Agency 1 were referred for other services within the agency at the end of the visit. Nevertheless, these data suggest that a sizable percentage of all families at each CYMH agency availed themselves of MHWCs, suggesting that this is a valuable service to maintain.

In terms of the number of MHWC visits, the median and mode were 1 for both agencies, despite the fact that 6.75 years of data were available from Agency 1 versus 3.66 years for Agency 2. About two–thirds of families had only one MHWC visit, suggesting that this service is meeting the needs of a majority of clients in the manner it was intended; namely, treating the session as if it will be the only session families have.

The second objective was to document how often and how soon families returned for a second MHWC and identify correlates of time to a second MHWC visit. About a third of families had two or more visits (32.3% Agency 1, 36.3% Agency 2). A fairly small percentage (3.6% Agency 1; 8.9% Agency 2) had four or more MHWC visits. This is generally in agreement with previous findings of single session services where 44%–50% of clients find a single session addressed their concerns.^7–10,17,41^ Future research is needed to understand reasons why some families have four or more visits. If families are waiting for more intensive services, repeated MHWC visits may be a way of managing while waiting. But it may also reflect difficulties engaging in treatment (e.g. scheduling conflicts), if families are receiving services but also using MHWCs. The range, mean, and median time to re-access was longer in Agency 1 compared to Agency 2. This may be, partly, because Agency 1 uses it as a point of intake and so families may be going on to other agency services. For Agency 1, the median time to a second visit was about 8.5 months; such a long time to a second visit would suggest families’ needs were likely met with a single session. In contrast, the median time for Agency 2 was 21 days; this fairly rapid return would suggest families’ needs were not met. Examining MHWC in relation to use of other services within CYMH agencies could help understanding how often MHWC may be supplementing other services. A qualitative study may be particularly useful to understand differing perspectives on when and why families use MHWC more than once.

Correlates for time to a second MHWC visit were few and only statistically significant for Agency 1. Of note, these differences remained even when the model for Agency 1 was run without disposition a discharge (see Supplemental Table S4). Younger children, children in shared custody or under the guardianship of their birth/adoptive mother or father, and children who were not referred to other agency services had higher risk of a second MHWC visit. Supplementary analyses showed that these children generally had more parenting and family problems. It is possible that parenting and family problems are causing significant impairment and/or burden and, thereby, need more supports. Unfortunately, information about severity, impairment, and burden was not available to test this hypothesis. Also, knowing if families were waiting for other services might also be informative, although such data are rarely captured concurrently within administrative datasets.

### Implications, strengths, and limitations

Out of all families who received services during the study window, a high percentage (24%, 61%) used the MHWC service. Even discounting that Agency 1 also used their MHWC as an intake, this suggest that the convenience of this service appeals to many families.

Of families using MHWCs, the majority have only one session while about a third have two or more sessions. This service delivery model can save families time, and agencies both time and money by eliminating the need to complete a detailed intake and/or assessment for cases that would go on to have a single visit within this service. Moreover, MHWCs can be tailored by agencies to meet their mandate and needs of their community. For example, agencies can use it as a point of intake for other services and as a point of referral for other agency services.

The time to a second MHWC varied considerably between the two agencies. As noted above, return for second MHWC visit within 3 weeks (median for Agency 2) may indicate the first visit was not sufficient. More research is needed to understand factors that may account for different patterns of MHWC visit use. The variation between agencies highlights the need for individual agency to examine their own data to better understand their own services are being used.

To the best of the authors’ knowledge, this is the first study to explore how MHWCs are used by families. This was done using electronic administrative data, which circumvents issues of recall associated with self-report measures and of attrition related to follow-up surveys/interviews. There were some limitations that are, however, important to note. First, there was a substantial list of inclusion and exclusion criteria. As such, the results are only generalizable to agencies similar to those that were recruited. One of the criteria required that agencies be located in a census division with a small urban center. Use of MHWCs may be different in agencies located in other census divisions. It is possible that, in larger urban centers, families have an MHWC visit at one agency and then other visits at another agency. Thus, time to a second visit at the first agency would be different, due to seeking help elsewhere. Of note, in this study, only 0.04% of families were referred to another service in the community. Another of the criteria required that agencies serve most mental health problems. The use of MHWCs may vary in agencies that specialize in some presenting concerns, like addictions. It is difficult to say, however, exactly how this and other criteria (e.g. age) would have affected the results.

Second, the agencies recorded the presenting concerns using a list of options. This approach is efficient, economical, and convenient for clinical use. However, using a validated measure with subscales would provide valuable information about the severity/impairment of problems, and how it compares to other children that age. This could be done by using measures that are publicly available to reduce the financial burden for agencies (e.g. Strengths and Difficulties Questionnaire).^42^

Third, children above 12 years old must consent to have a file opened for them. This means that files are not opened (i.e. no information is recorded) when a parent of a child above 12 years old accesses an MHWC *without* their child present. As such, these families could not be included or accounted for. Fourth it is difficult to determine why there were differences in time to a second visit and correlates of this outcome given that there are number of factors by which the agencies differ. Some of these factors (e.g. scheduled vs unscheduled, intake vs single session) could not be explored because the agencies do not routinely enter these data into the system. Fifth, the sample size in Agency 2 was significantly smaller, which may have limited the power to detect significant relationships and influenced the overall model fit. Finally, data on other agencies or health/mental health services was not available. It possible that children accessed MHWCs from another agency, family physician, or other health care provider, which were not captured in this study.

### Future studies

There are, at least, three crucial areas for future research. First, exploring how MHWCs fit in with other services provided by CYMH agencies (e.g. MHWCs used exclusively, MHWCs alongside other agency services). Second, assessing the effectiveness of MHWCs. There is a paucity of research in this area, likely because of the challenges with conducting these studies (e.g. attrition with follow-up studies). A systematic, province-wide implementation of MHWCs would be very useful to evaluate the effectiveness of this service delivery model. This implementation could incorporate the use of brief, standardized outcome measures assessing psychopathology, impairment, and burden. Other key information could also be recorded (e.g. scheduled vs unscheduled sessions, other service use). These variables are important but could not be explored in the current dissertation as they are not routinely collected and recorded by CYMH agencies. Finally, exploring patterns of service use across sectors by linking administrative data to Ontario Health Insurance Plan (OHIP) data. There may be a group of families that are accustomed to using unscheduled or walk-in services, whether for health or mental health concerns, and which may be distinguished from other families.

## Conclusion

Many CYMH agencies across Canada and in Ontario are using MHWCs. This is a promising model of service delivery, showing perceived improvement immediately after the MHWC session as well as at follow–up. There is a paucity of research examining how MHWCs are used by families and correlated of its use. Overall, about a third of families using MHWCs had two or more visit. Child age, guardianship, and disposition at discharge emerged as correlates of time to a second MHWC visit.

## Supplemental Material

sj-docx-1-map-10.1177_27550834231200617 – Supplemental material for Accessing and re-accessing mental health walk-in clinics for children and families Click here for additional data file. Supplemental material, sj-docx-1-map-10.1177_27550834231200617 for Accessing and re-accessing mental health walk-in clinics for children and families by Catalina Sarmiento and Graham J. Reid in The Journal of Medicine Access

## Declarations

**Ethics approval and consent to participate:** The study received ethics approval by The University of Western Ontario Research Ethics Board (approval id. 115545). The participating agencies signed agreements specifying the data that would be provided for the project and dissemination of findings.

**Consent for publication:** Not applicable.

**Availability of data and materials:** The participating agencies did not consent for their data to be shared publicly, so supporting data are not available.
