# Supplementary material for: Accessing and re-accessing mental health walk-in clinics for children and families
Source: J Med Access. 2023 Sep 29;7:27550834231200617. doi: 10.1177/27550834231200617 (PMC10541731; doi:10.1177/27550834231200617)
Supplement: sj-docx-1-map-10.1177_27550834231200617 – Supplemental material for Accessing and re-accessing mental health walk-in clinics for children and families [file sj-docx-1-map-10.1177_27550834231200617.docx]

Supplemental Materials

**Table S1.** Disposition at discharge subcategories after first MHWC visit for all families with a MHWC visit.

|  | Agency 1  (N=2540) |
| --- | --- |
|  | n (%) |
| No referral within the agency | 1517 (59.7%) |
| Mutual completion | 745 (29.3%) |
| No referral | 771 (30.4%) |
| Referral to external agency | 1 (0.04%) |
| Referral within the agency | 961 (37.8%) |
| Still waiting for another service | 5 (0.2%) |
| Referral within the agency | 945 (37.2%) |
| Wanting additional services and directed to intake | 11 (0.4%) |
| Missing | 62 (2.4%) |

**Table S2.** Analyses of missing data for the Cox regression models predicting time to a second MHWC visit.

| Total missing across correlates | Agency 1 | Agency 2 |
| --- | --- | --- |
|  | n (%) | n (%) |
| No missing data | 1740 (69.0%) | 293 (68.0%) |
| 1 | 322 (12.8%) | 107 (24.8%) |
| 2 | 434 (17.2%) | 17 (3.9%) |
| 3 | 24 (0.9%) | 3 (0.7%) |
| 4 | 1 (0.04%) | 2 (0.5%) |
| 5 | - | 9 (2.1%) |

*Note.* Analyses by families/cases (e.g., 12.8% of families were missing data on 1 correlate for Agency 1).

**Table S3.** Number of MHWC visits that families had.

|  | Agency 1  (N=2540) | Agency 2  (N=433) |
| --- | --- | --- |
|  | n (%) | n (%) |
| 1 | 1720 (67.7%) | 276 (63.7%) |
| 2 | 540 (21.3%) | 92 (21.2%) |
| 3 | 188 (7.4%) | 27 (6.2%) |
| 4 | 53 (2.1%) | 16 (3.7%) |
| 5 | 23 (0.9%) | 11 (2.5%) |
| 6 | 9 (0.4%) | 4 (0.9%) |
| 7 | 3 (0.1%) | 4 (0.9%) |
| 8 | 1 (0.04%) | 3 (0.7%) |
| 9 | 2 (0.1%) | - |
| 10 | 1 (0.04%) | - |

**Table S4.** Unadjusted and adjusted hazards ratios for time to a second visit without disposition at discharge.

|  | Agency 1 | |  | Agency 2 | |
| --- | --- | --- | --- | --- | --- |
|  | Unadjusted | Adjusted |  | Unadjusted | Adjusted |
|  | HR (95% CI) | HR (95% CI) |  | HR (95% CI) | HR (95% CI) |
| Social content |  |  |  |  |  |
| Child age ^a^ |  |  |  |  |  |
| <12 years old | 1.18  (1.02-1.37)* | 1.20  (1.03-1.39)* |  | 1.20  (0.86-1.67) | 1.21  (0.85-1.72) |
| Child gender ^b^ |  |  |  |  |  |
| Male | 1.04  (0.90-1.19) | 1.02  (0.88-1.18) |  | 0.79  (0.57-1.11) | 0.75  (0.52-1.07) |
| Neighbourhood poverty ^c^ |  |  |  |  |  |
| High poverty | 1.17  (1.01-1.36)* | 1.14  (0.97-1.33) |  | 0.84  (0.60-1.18) | 0.81  (0.57-1.14) |
| Guardianship of child ^d^ |  |  |  |  |  |
| Shared custody | 1.32  (1.07-1.63)* | 1.32  (1.05-1.66)* |  | 1.20  (0.71-2.03) | 1.23  (0.72-2.10) |
| Birth/adoptive mother or father | 1.29  (1.07-1.55)** | 1.29  (1.06-1.58)* |  | 0.85  (0.58-1.25) | 0.83  (0.55-1.24) |
| Other | 1.22  (0.90-1.65) | 1.24  (0.89-1.71) |  | 1.01  (0.55-1.86) | 1.02  (0.54-1.91) |
| Presenting concern |  |  |  |  |  |
| Externalizing ^e^ | 0.98  (0.82-1.18) | 0.97  (0.75-1.24) |  | 0.94  (0.67-1.32) | 1.04  (0.52-2.08) |
| Internalizing ^e^ | 0.99  (0.84-1.19) | 1.03  (0.81-1.32) |  | 0.99  (0.71-1.41) | 1.05  (0.54-2.05) |
| Parenting and family ^e^ | 1.01  (0.84-1.21) | 0.96  (0.74-1.25) |  | 0.96  (0.59-1.56) | 1.04  (0.51-2.12) |
| Other ^e^ | 0.97  (0.80-1.18) | 0.99  (0.76-1.29) |  | 1.03  (0.73-1.46) | 1.02  (0.52-1.99) |
| Number of presenting concerns ^f^ |  |  |  |  |  |
| 1 | 1.04  (0.80-1.35) | 1.07  (0.68-1.68) |  | 1.13  (0.71-1.79) | 1.22  (0.34-4.34) |
| 2 | 1.04  (0.81-1.34) | 1.03  (0.75-1.42) |  | 1.28  (0.79-2.07) | 1.39  (0.64-3.05) |

* *p* <.05 ** *p* <.01

^a^ Reference category is children 12+

^b^ Reference category is females.

^c^ Reference category is low poverty.

^d^ Reference category is birth/adoptive parents.

^e^ Reference category is no presenting problem in that category.

^f^ Reference category 3+ presenting concern categories.

**Table S5.** Descriptive statistics of child, family, and service use for families included in the Cox regression, following multiple imputation.

|  | Agency 1 | Agency 2 |
| --- | --- | --- |
|  | n (%) | n (%) |
| ***Child*** |  |  |
| Child age |  |  |
| <12 years old | 1601 (63.5%) | 254 (60.2%) |
| 12+ years old | 920 (36.5%) | 168 (39.8%) |
| Child gender |  |  |
| Female | 1272.3 (50.5%) | 190.5 (45.1%) |
| Male | 1248.7 (49.5%) | 231.6 (54.9%) |
| ***Family*** |  |  |
| Guardianship of child |  |  |
| Birth/adoptive parents | 1001.7 (39.7%) | 181.7 (43.1%) |
| Shared custody | 494.9 (19.6%) | 53.8 (12.7%) |
| Birth/adoptive mother/father | 845.2 (33.5%) | 149.3 (35.4%) |
| Other | 179.2 (7.1%) | 37.3 (8.8%) |
| Neighborhood poverty |  |  |
| Low poverty | 1786 (70.8%) | 259.4 (61.5%) |
| High poverty | 735 (29.2%) | 162.7 (38.5%) |
| ***Service use*** |  |  |
| Presenting concern^1^ |  |  |
| Externalizing | 1166.2 (46.3%) | 188.7 (44.7%) |
| Internalizing | 1594.3 (63.2%) | 253.8 (60.1%) |
| Parenting and family | 992.1 (39.4%) | 75.2 (17.8%) |
| Other | 799.9 (31.7%) | 255.5 (60.5%) |
| Number of presenting concerns |  |  |
| 1 | 1102.8 (43.7%) | 178.2 (42.2%) |
| 2 | 898.4 (35.6%) | 143.2 (33.9%) |
| 3+ | 519.9 (20.6%) | 100.7 (23.9%) |
| Disposition at discharge |  |  |
| No referral within the agency | 1558.5 (61.8%) | N/A |
| Referral within the agency | 962.5 (38.2%) | N/A |

^1^ Clinicians could code multiple presenting concerns for a visit.

###### **Table S6a.** Relationship between presenting concerns and child age for Agency 1.

|  | Child Age | |
| --- | --- | --- |
|  | <12 years old | 12+ years old |
| Externalizing | 52.1% | 36.0%* |
| Internalizing | 59.3% | 70.1%* |
| Parenting and family | 40.7% | 37.1% |
| Other | 30.2% | 34.5% |

* *p* < .01.

*Note.* The table summarizes the percentages of families with a presenting concern category within an age group. Column percentages are reported; as families could have had more than one presenting concern, percentages do not sum to 100. For example, 52.1% of children <12 years old presented with externalizing problems, whereas 36.0% of children 12+ year old presented with externalizing problems. The difference in externalizing problems between <12 year old and 12+ year old groups (i.e., 52.1% vs. 36.0%) is statistically significant, which is denoted by the asterisk.

###### **Table S6b.** Relationship between presenting concerns and guardianship for Agency 1.

|  | Guardianship | | |
| --- | --- | --- | --- |
|  | Birth/adoptive parents | Shared custody | Birth/adoptive father/mother |
| Externalizing | 45.0% | 42.2% | 48.4% |
| Internalizing | 66.6% | 62.5% | 60.4%* |
| Parenting and family | 26.2% | 53.1%* | 44.2%* |
| Other | 32.3% | 28.7% | 32.9% |

* *p* < .01.

*Note.* The table summarizes the percentages of families with a presenting concern category within a guardianship group. Column percentages are reported; as families could have had more than one presenting concern, percentages do not sum to 100. Comparisons are made to children under the guardianship of both parents because this was the reference category. For example, 66.6% percent of children under the guardianship of both parents presented with internalizing problems, 62.5% of children in shared custody presented with internalizing problems, and 60.4% of children under the guardianship of father/mother presented with internalizing problems. The difference in internalizing problems between children under the guardianship of both parents is statistically significant compared to children under the guardianship of father/mother (i.e., 66.6% vs. 60.4%) which is denoted by the asterisk, but not compared to children in shared custody (i.e., 66.6% vs. 62.5%).

###### **Table S6c.** Relationship between presenting concerns and disposition at discharge for Agency 1.

|  | Disposition at discharge | |
| --- | --- | --- |
|  | No referral within the agency | Referral within the agency |
| Externalizing | 44.4% | 49.2% |
| Internalizing | 57.5% | 72.6%* |
| Parenting and family | 42.2% | 34.8%* |
| Other | 33.3% | 29.1% |

* *p* < .01.

*Note.* The table summarizes the percentages of families with a presenting concern category within a disposition at discharge group. Column percentages are reported; as families could have had more than one presenting concern, percentages do not sum to 100. For example, 57.5% of children with “no referral within the agency” presented with internalizing problems, whereas 72.6% of children with “referral within the agency” presented with internalizing problems. The difference in internalizing problems between “no referral within the agency” and “referral within the agency” groups (i.e., 57.5% vs. 72.6%) is statistically significant which is denoted by the asterisk.

**Figure S1.** Survival probability by time. Survival, in the y axis, means not having a second MHWC visit. Time, in the axis, is measured in days.
